# Parental divorce in late adolescence does not seem to increase mental health problems: a population study from Norway

**DOI:** 10.1186/1471-2458-13-413

**Published:** 2013-04-30

**Authors:** Henok Zeratsion, Madeleine Dalsklev, Espen Bjertness, Lars Lien, Ole R Haavet, Jon A Halvorsen, Cecilie B Bjertness, Bjørgulf Claussen

**Affiliations:** 1 Institute of Health and Society, University of Oslo, Box 1130, Blindern, Oslo 0318, Norway; 2 Institute of Psychology, University of Oslo, Box 1130, Blindern, Oslo 0318, Norway; 3 National Center for Dual Diagnoses, Innlandet Hospital Trust HF 2312, Ottestad, Norway; 4 Department of General Practice, Institute of Health and Society, University of Oslo, Blindern, Norway; 5 Department of Dermatology, Oslo University Hospital Rikshospitalet, Faculty of Medicin, Blindern, Norway; 6 Tibet University Medical College, Lhasa, Tibet, China

**Keywords:** HSCL, SDQ, Adolescents, Internalized and externalized mental health problems, Mental distress, Parental divorce

## Abstract

**Background:**

Former studies have shown increased mental health problems in adolescents after parental divorce all over the Western world. We wanted to see if that still is the case in Norway today when divorce turns to be more and more common.

**Methods:**

In a prospective study design, two samples were constituted, adolescents at a baseline survey in 2001/02 (n = 2422) and those at follow-up in 2003/04 (n = 1861), when the adolescents were 15/16 and 18/19 years-old, respectively. They answered self-administered questionnaires in both surveys of Young-HUBRO in Oslo. Early parental divorce was defined as that which occured before age 15/16 years, and late divorce occured between age 15/16 and 18/19. Internalized and externalized mental health problems were measured by the Hopkin’s Symptom Check List (HSCL-10) and the Strengths and Difficulties Questionnaire (SDQ).

**Results:**

After linear regression models were adjusted for gender, ethnicity, family economy, social support, and mental health problem symptoms measured at baseline before parental divorce occured, late parental divorce did not lead to significant increase in mental health problems among adolescents in the city of Oslo. Early parental divorce was associated with internal mental health problems among young adolescents when adjusted only for the first four possible confounders.

**Conclusions:**

It seems that parental divorce in late adolescence does not lead to mental health problems in Norway any more, as has been shown before, while such problems may prevail among young adolescents. This does not mean that parental divorce create less problems in late adolescence than before but these youths might have developed adjustment abilities against health effects as divorce have turned to be more common.

## Background

Mental health problems in children and adolescents after parental divorce have been regularly reported during the last decades [1]. A meta-analysis from 1991 showed that the effect sizes were on a decreasing trend as divorce became more common [2] but still this problem seems to have increased in the 1990s as some social forces have operated more strongly to disadvantage children from divorced families [1].

A Norwegian longitudinal study from the 1990s in Nord-Trøndelag county by Størksen et al. found that adolescents aged 13 to 19 years who experienced parental divorce had higher levels of anxiety and depression than others [3,4]. Studies from Finland found that mental distress after parental divorce prevailed into adulthood [5,6], and a recent longitudinal study from Finland by Fröjd et al. also found that adolescents who experienced change in the caretaking parent had increased internalized symptoms when compared with those who did not experience caretaker change or moved away from home [7].

Studies of demographic factors regularly find that internalized symptoms like depression and anxiety generally increase with age among adolescents [3,8] while Størksen et al. [3] showed that young adolescents of divorced parents had higher mental distress beyond that observed in the general trend. Studies of gender differences have shown mixed results. Dutch girls reported more mental distress symptoms after parental divorce than boys [9], while an American study found the opposite result [10], and other studies have found a similar association among boys and girls [1].

Various family constellations have different effects on adolescents’ mental health [7,11]. Offsprings of single parents tend to have higher mental distress than adolescents living with both parents [12]. The legal and social differences between cohabiting and married couples are small in Scandinavia today; the most marked difference is that cohabitants have a two to three times higher risk of separation [13].

The objectives of the present longitudinal study were 1) to study if internalized and externalized mental health problems were higher among adolescents who experienced early parental divorce when compared with their 15/16 years-old peers from continuously married parents, and 2) to investigate if such mental health problems were higher among adolescents who experienced late parental divorce when compared with their 18/19 years-old peers whose parents were still married.

## Methods

This was a cohort study where all 15/16 years-old, 10th grade students in the city of Oslo (n = 4273) were invited to participate in the baseline survey of Young-HUBRO in 2000/2001 [14]. The response rate was 89% (n = 3811).

Self-administered questionnaires were mostly answered in class room sessions; for 13% of the participants the questionnaire was sent by post at the follow-up because they did not attend school any more. Those who stated parents’ marital status was other than married/cohabitants or divorced/separated (n = 191) and those who gave contradictory answers in the two surveys (n = 55) were no more considered for our purpose, and were excluded from the participants in the first survey of Young-HUBRO (n = 3811), giving a new sample of 3565. Of these, 1076 did not participate at the second survey and 67 did not respond to the question on marital status, giving the final baseline sample of 2422 in the present study. The sample at follow-up (n = 1861) was determined after exclusion of those who experienced early parental divorce (n = 530) and those whose parents’ marital status was other than married/cohabitants or divorced/separated at the second survey (n = 31).

### Dependent variable: mental health problems

*The Hopkin’s Symptom Check List* (HSCL-10) was used to measure internalized mental health problems. Ten questions were asked to find out how troubled the participants were by depression and anxiety symptoms during the previous week. Four possible answers scored from 1 (not troubled) to 4 (heavily troubled) gave a continuous variable with score range from 1 to 4.

The *Strengths and Difficulties Questionnaire* (SDQ externalized) was used to measure externalized mental health problems that include hyperactivity and conduct symptoms. Considering their own experience in the last six months, the adolescents were asked to rate each of the 10 items by stating one of three possible answers, “not true” (0), “somewhat true” (1) or “certainly true” (2), giving scores from 0 to 20. We combined the hyperactivity and conduct problems into one score, as in other Young HUBRO studies (14,15). The internal reliability using Cronbach’s alpha for HSCL-10 was 0.88 and for SDQ 0.67.

### Independent variables

*Late parental divorce* that occurred between 2001 and 2004 (n = 109) and parental divorce reported in 2001, which was referred to as *early parental divorce* (n = 530), were our main independent variables. Parental divorce was defined by a question about parents’ marital status: “My parents are: 1) married/cohabitants, 2) unmarried, 3) divorced/separated, 4) one or two are dead, 5) other”. The adolescents’ self-report of biological parents’ split-up in the form of divorce or separation constituted the term “parental divorce”. Adolescents having divorced/separated parents were compared at the same point of time with those whose parents were married or cohabiting partners.

Potential confounders include gender, ethnicity, family economy and social support. These variables are found to be associated with mental distress in other studies [8,15], and should be adjusted for.

*Ethnicity* was dichtomized into “western” and “non-western” based on parents’ place of birth according to the definitions of Statistics Norway. *Family economy* was dichtomized into “above average” and “average and below”, based on the question “I believe, relative to others in Norway, my family has: 1) ‘poor economy’, 2) ‘average economy’; 3) ‘good economy’; or 4) ‘very good economy’.” *Social support* was created by summarizing the response of two questions that focused on availability of help: “How many persons outside your immediate family are so close to you that you can rely on to get help 1) if you have personal problems, 2) if you have practical problems (for example, school assignments)?” Those who answered “0” or “1” for each of these two separate questions formed the *low social support* category and the rest were grouped to form the *high social support* category. This gave a cut-off point at 85th and 78th percentile in the first and the second question, respectively. Cronbach’s alpha was 0.57, and mean inter-item correlation value was 0.42 which is within an optimal range for a scale with fewer than 10 items [16]. The number of people to whom the adolescents can talk and rely on for help when they have personal or practical problems was used as a proxy measure for social support which is also used in other studies (8).

### Missing values

We included in the baseline sample those who participated in both surveys and answered the main questions, giving missing values of 32% of the 3565 respondents in the first Young-HUBRO survey. 42% of non-western and 27% of western adolescents were non-respondents. Non-response was random at the other independent variables.

### Statistics

Pearson’s chi square test was used to study bivariate associations (Table 1), and paired samples t-test for difference in mental distress scores (Table 2). Multiple linear regressions were analysed for early and late parental divorces separately using HSCL-10 and SDQ scores as independent variables (Table 3). Potential confounding variables were gender, ethnicity, family economy, and social support measured at baseline. At follow-up, the regression models were controlled for baseline HSCL-10 and SDQ scores rendering a prospective study design.

**Table 1:** **Late parental divorce and early parental divorce** (**horizontal percentages**) **across other independent variables at the first survey** (**vertical percentages**)

	**Girls**			**Boys**		
**Independent variables**	**Frequency (Vertical percentage)**	**Late divorce**	**Early divorce**	**Frequency (Vertical percentage)**	**Late divorce**	**Early divorce**
Ethnicity						
Western	1169 (87.2)	54 (4.6)	275 (23.5)**	963 (89.9)	39 (4.0)	221 (22.9)**
Non western	172 (12.8)	10 (5.8)	24 (14.0)	108 (10.1)	6 (5.6)	7 (6.5)
Family economy						
Average and below	390 (29.6)	20 (5.1)	131 (33.6)**	265 (24.8)	19 (7.2)*	95 (35.8)**
Above average	927 (70.4)	42 (4.5)	165 (17.8)	803 (75.2)	26 (3.2)	136 (16.9)
Social support						
Low	118 (8.8)	5 (4.2)	30 (25.4)	95 (8.9)	3 (3.2)	23 (24.2)
High	1219 (91.2)	59 (4.8)	269 (22.1)	978 (91.1)	42 (4.3)	208 (21.3)

Significant difference at **(*p* <0.01) and at *(*p* < 0.05) between the categories of an independent variable under the same type of parental divorce.

**Table 2:** **Mean test scores at two points of time across parental divorce categories** (**95**% **confidence interval**)

	**Girls**					**Boys**				
		**HSCL**-**10**		**SDQ externalized**			**HSCL**-**10**		**SDQ externalized**	
**Parental divorce**	**n**	**2001**	**2004**	**2001**	**2004**	**n**	**2001**	**2004**	**2001**	**2004**
Late divorce	64	1.66 (1.53-1.80)	1.76 (1.61-1.91)	5.59.(5.00-6.17)	5.94 (5.39-6.77)	45	1.39 (1.26-1.52)	1.54 (1.38-1.70)*	6.44 (5.43-7.45)	5.84 (4.98-6.71)
Early divorce	299	1.69 (1.62-1.75)	1.80 (1.73-1.87)*	5.88 (5.56-6.19)	6.01 (5.70-6.34)	231	1.34 (1.29-1.39)	1.39 (1.34-1.45)	5.28 (4.87-5.70)	5.11 (4.76-5.51)
Cont. married	959	1.58 (1.55-1.61)	1.70 (1.67-1.74)*	5.28 (5.11-5.45)	5.31 (5.14-5.48)	793	1.28 (1.26-1.31)	1.35 (1.32-1.38)*	5.21 (5.00-5.43)	4.90 (4.70-5.10)*

*P < 0.01 for the difference in test scores between the two time points for the same divorce category.

**Table 3:** **Internalized and externalized mental health problems among 15**/**16 and 18**/**19 years**-**old adolescents at baseline and follow**-**up**, **across parental divorce** (**regression coefficients**)

		**Baseline results (n** = **2422)**		**Follow**-**up results (n** = **1861)**	
	**Explanatory variables**	**HSCL-10 (CI:95%)**	**SDQ (CI:95%)**	**HSCL-10 (CI:95%)**	**SDQ (CI:95%)**
Crude results	Parental divorce*	0.84 (0.36 – 1.32)	0.31 (0.03 – 0.59)	0.56 (−0.35 – 1.46)	0.42 (−0.04 – 0.88)
Adjusted results	Parental divorce*	0.47 (0.02 – 0.92)	0.15 (−0.14 – 0.43)	0.50 (−0.38 – 1.39)	0.38 (−0.08 – 0.85)
	(ref = continuously married)				
	Gender (ref = boys)	2.99 (2.62 - 3.35)	0.11 (−0.12 – 0.34)	1.86 (1.42 – 2.30)	0.36 (0.14 – 0.58)
	Ethnicity (ref = western)	0.14 (−0.44 – 0.73)	−0.28 (−0.65 – 0.10)	0.64 (0.02 – 1.27)	−0-03 (−0.36 – 0.30)
	Social support (ref = low)	−3.58 (−4.23 - -2.94)	−1.54 (−1.95 - -1.13)	−1.44 (−2.21 - -0.66)	0.06 (−0.34 – 0.46)
	Family economy	−1.42 (−1.84 - -1.00)	−0.58 (−0.85 - -0.31)	−0.53 (−1.04 - -0.02)	−0.19 (−0.46 – 0.07)
	(ref = average & below)				

*Parental divorce refers to early divorce at baseline and late divorce at follow-up.

### Ethics

Informed consent was given by students and parents. The study was approved by the regional ethical committee for medical research.

## Results

Early parental divorce was reported by 22.3% of girls and 21.4% of boys, late divorce by 6.3% and 5.4%, respectively. Western adolescents reported more early divorces than non-western, and so did adolescents from families with average or below average economy (Table 1). Among boys, late parental divorce was also associated with family economy.

After a late parental divorce, HSCL-10 score increased significantly among boys between baseline and follow-up, while among girls the HSCL-10 score increased significantly among those who experienced early divorce (Table 2). Both girls and boys from continuously married parents were also observed to have their HSCL-10 score significantly increased between baseline and follow-up. SDQ scores increased among boys from continuously married parents.

Crude regression results showed that early parental divorce was associated with internalized and externalized mental health problems at age 15/16 years (Table 3). After adjustment, the association of early parental divorce with internalized mental health problems sustained significant, while the association with externalized mental health problems turned to be non-significant. Late parental divorce was not associated with either internalized or externalized mental health problems at follow-up.

## Discussion

We found that *late* parental divorce which occurred when the adolescents were between 15 and 19 years old had no influence on internalized and externalized mental health problems. *Early* parental divorce that occurred before the age of 15, on the other hand, was found to have significant association with internalized mental health problem at the baseline survey.

The strength of the study is its prospective design based on a cohort of adolescents aged 15/16 years living in an urban area with diverse cultural, social and economic background. At follow-up, we could compare change in mental distress that ocurred between baseline and follow-up among adolescents who experienced late parental divorce with the change among those whose parents were still married. A limitation is the small sub-sample of those who experienced late parental divorce with risk of type 2 errors. A 32% loss-to-follow-up may influence estimates of prevalence but association measures are shown to be robust to attrition in this sample [14]. Since non-response was common among non-western students our estimates are more unsecure for ethnic non-Norwegians. We have used self-reported mental health symptoms which are well validated [17,18] but self-reported symptoms are arguable in epidemiological research, especially in children and adolescents [7].

Our results that show association of *early* parental divorce with internalized mental health problems are supported by earlier Norwegian [4,11] and other western [19,20] studies. In the former Norwegian study from Nord-Trøndelag county Størksen et al. found about the same increase in mental distress despite that the divorce rate in their mostly rural area is half of that of Oslo [3,21]. They had the objectives, among others, of determining whether the effect of *early* divorce was different at follow-up compared to baseline [1]. We have not found any prospective study like ours of mental health problems after *late* parental divorce but our results seems to differ from earlier Scandinavian [6,22,23] and western studies [1,19,20]. We find no increase in mental health problems among adolescents who experienced late parental divorce compared to their 18/19 years-old peers whose parents were still married at follow-up. Two of these three Finish and Swedish studies had data on children and adolescents from about twenty years ago. Thus, the effects of parental distress in late adolescence may have diminished in recent years.

Fröjd et al. recent study from Finland used another method, showing significant influence of *caretaker change* on depression [7]. We have similar data of caretaker changes, and found the same. In both studies, caretaker change refers to various forms of family transitions including parental divorce. Interestingly, adolescents moving away from home did not show significant changes in internalizing problems in both studies. This only recent study from Scandinavia does not contradict our results.

Another possible interpretation of these results may be that our measures of mental health problems are not good enough; however, our two tests are well validated and much used [17,18]. A type 2 error because of a small sample is a possibility; in that case the effect among these adolescents in Oslo probably are smaller than found before.

We find it most probable that internalized (anxiety and depression) and externalized (conduct) health problems after parental divorce in late adolescence are not higher any more compared to children of non-divorced parents. This does not mean that parental divorce is not followed by many problems for children and adolecents, both emotional and practical burdens. Our measures are directed towards mental disease-related problems, and they may have been reduced as divorce has turned more common. Other problems of the offsprings after divorce may still be overwhelming, and should get much concern among parents and others taking care of children and adolescents.

## Conclusion

While *early* parental divorce was associated with internalized mental health problems, we do not find such effects of late divorce on adolecents aged 18/19 years in an urban sample. This may mean that our methods are not good enough, or that the adjustment abilities of adolescents of divorce have improved. This does not mean that problems for the offsprings after divorce have become less than in former times.

## Competing interests

The authors declare that they have no competing interests.

## Authors’ contributions

All authors decided design and methods, HZ did the analyses and wrote the manuscript drafts, all authors read and approved the final manuscript, and BC supervised the work.

## Pre-publication history

The pre-publication history for this paper can be accessed here:

http://www.biomedcentral.com/1471-2458/13/413/prepub
